# Modeling Resilience to Damage in Multiple Sclerosis: Plasticity Meets Connectivity

**DOI:** 10.3390/ijms21010143

**Published:** 2019-12-24

**Authors:** Mario Stampanoni Bassi, Ennio Iezzi, Luigi Pavone, Georgia Mandolesi, Alessandra Musella, Antonietta Gentile, Luana Gilio, Diego Centonze, Fabio Buttari

**Affiliations:** 1 Unit of Neurology & Neurorehabilitation, IRCCS Neuromed, 86077 Pozzilli (IS), Italy; m.stampanonibassi@gmail.com (M.S.B.); ennio.iezzi@neuromed.it (E.I.); gi19gi82@gmail.com (L.P.); gilio.luana@gmail.com (L.G.); fabio.buttari@gmail.com (F.B.); 2 Laboratory of Synaptic Immunopathology, IRCCS San Raffaele Pisana, 00163 Rome, Italy; georgia.mandolesi@sanraffaele.it (G.M.); alessandra.musella@sanraffaele.it (A.M.); gntnnt01@uniroma2.it (A.G.); 3 Laboratory of Synaptic Immunopathology, San Raffaele University of Rome, 00166 Rome, Italy; 4 Laboratory of Synaptic Immunopathology, Department of Systems Medicine, Tor Vergata University, 00173 Rome, Italy

**Keywords:** synaptic plasticity, long-term potentiation (LTP), synaptic scaling, brain networks, inflammation, connectivity, multiple sclerosis, resting state functional MRI (rs-fMRI)

## Abstract

Multiple sclerosis (MS) is a chronic inflammatory disease of the central nervous system (CNS) characterized by demyelinating white matter lesions and neurodegeneration, with a variable clinical course. Brain network architecture provides efficient information processing and resilience to damage. The peculiar organization characterized by a low number of highly connected nodes (hubs) confers high resistance to random damage. Anti-homeostatic synaptic plasticity, in particular long-term potentiation (LTP), represents one of the main physiological mechanisms underlying clinical recovery after brain damage. Different types of synaptic plasticity, including both anti-homeostatic and homeostatic mechanisms (synaptic scaling), contribute to shape brain networks. In MS, altered synaptic functioning induced by inflammatory mediators may represent a further cause of brain network collapse in addition to demyelination and grey matter atrophy. We propose that impaired LTP expression and pathologically enhanced upscaling may contribute to disrupting brain network topology in MS, weakening resilience to damage and negatively influencing the disease course.

## 1. Introduction

Multiple sclerosis (MS) is a chronic inflammatory disease of the central nervous system characterized by demyelination and neurodegeneration. The clinical course of MS is highly variable and different clinical phenotypes have been described. While the introduction of MRI significantly improved MS diagnosis [1], a discrepancy between radiological findings and clinical manifestations is frequently observed [2]. Different mechanisms contribute to this “clinico-radiological paradox” and both synaptic plasticity and brain networks architecture may play an important role, influencing resilience to damage.

Several studies have demonstrated that even in the absence of the associated visible damage, inflammation in MS negatively affects the disease course [3,4]. It has been shown indeed that in animal models (i.e., experimental autoimmune encephalomyelitis, EAE) and in MS patients, specific proinflammatory cytokines alter synaptic transmission and plasticity [5,6]. Therefore, subverted synaptic plasticity induced by inflammation may represent an independent cause of brain network dysfunction in MS [7].

In the present article, we will overview the alterations of synaptic plasticity and brain connectivity induced by inflammation in MS. We propose that impaired synaptic plasticity expression could contribute to disrupting brain network topology, critically weakening resilience to damage.

## 2. Clinico-Radiological Paradox, Brain Network Resilience and Plasticity

In different neurological conditions, brain networks are able to maintain appropriate functional efficiency even in the presence of a high structural damage load, delaying or minimizing the appearance of clinical symptoms [8]. A growing number of studies have demonstrated that the organization of brain networks is highly specialized and reveals specific features evolved to improve efficiency, containing the wiring cost. This architecture protects networks from random attacks and is useful for optimal reorganization after damage [8,9]. In addition, synaptic plasticity is one of the main physiological mechanisms involved in brain network remodeling and critically contributes to brain damage compensation. Experimental and clinical studies have clearly shown that the efficiency of synaptic plasticity mechanisms, particularly of long-term potentiation (LTP), influences the chances of recovery after damage [10,11].

### 2.1. Brain Network Architecture Provides Resilience to Damage

Graph theory offers a helpful approach to analyzing connectivity data deriving from neurophysiological (i.e., electroencephalography and magnetoencephalography) or imaging (i.e., functional MRI, fMRI) techniques [12,13,14,15,16,17]. Complex brain networks can be modeled as a series of nodes, indicating different brain regions, and edges, representing the connections between nodes. The edges are defined on the basis of structural (i.e., diffusion tensor imaging, DTI) or functional connectivity (i.e., temporal covariation of spontaneous activity) between different regions [17,18,19]. Specific measures describing network functioning have been defined. In particular, brain networks are characterized by elevated global and local efficiency, modularity and scale-free degree distribution [8]. It has been argued that some key features of brain networks directly arise from the pattern of connections between nodes. An important characteristic of brain network organization, supporting the segregation of information processing, is the presence of modules, defined as communities of highly connected nodes with relatively few connections with nodes of other modules [20]. Moreover, the presence of a small number of hubs, consisting of highly connected nodes with elevated centrality, is essential for the integration of information and to increase network efficiency [21]. Hubs are highly interconnected, between them forming a subgroup of regions, the rich club, which critically contribute to brain network functioning [15,21]. Hubs develop through a “rich get richer” rule, meaning that the preferential attachment of new nodes occurs with those that have higher degree [22]. This architecture is associated to strong resistance to random damage due to the numerical prevalence of low connected nodes [8,23]. Conversely, targeted damage to hubs critically alters network functionality as shown in different neurological conditions [24].

### 2.2. Synaptic Plasticity Enables Symptom Compensation

Synaptic plasticity is the ability of neurons to modify the strength of reciprocal connections and is a key neurophysiological mechanism involved in network development and reorganization after damage. Different forms of synaptic plasticity have been studied, including anti-homeostatic and homeostatic mechanisms [25,26]. LTP has been first explored in hippocampal neurons and consists of persistent enhancement of synaptic excitability associated with remodeling of the presynaptic and postsynaptic terminals [26,27].

LTP has been consistently associated to learning and memory [27]; in addition, LTP is critically involved in brain damage compensation [10]. In experimental models, it has been shown that synaptic rearrangement in the peri-lesional area after focal ischemic brain damage in rats is critical for clinical recovery. In particular, electrophysiological recordings have demonstrated that increased glutamatergic transmission in surviving neurons is correlated with the degree of clinical improvement [10]. The key role of LTP in the compensation of clinical manifestations has also been documented in humans. Accordingly, in patients with acute stroke, clinical recovery after six months was positively correlated with the efficiency of LTP-like plasticity mechanisms probed using transcranial magnetic stimulation (TMS), namely LTP reserve [11]. Moreover, in RR-MS patients, the magnitude of plasticity reserve measured in the acute phase was correlated with the clinical recovery that occurred three months later [28].

### 2.3. Synaptic Plasticity and Network Remodeling

*N*-methyl-d-aspartate receptors (NMDARs) are essential for inducing LTP [29,30]. Specific properties of this form of Hebbian plasticity, including cooperativity, associativity and input-specificity, are directly associated to NMDAR characteristics [31]. LTP is an anti-homeostatic phenomenon, as it is associated with increased synaptic excitability, which in turn further promotes LTP induction [25]. Another form of Hebbian, anti-homeostatic plasticity associated with NMDAR activation is long-term depression (LTD), which induces lasting reduction in synaptic excitability [25,31]. The characteristics of Hebbian plasticity, in particular of LTP, suggest that this mechanism might be involved in the generation of specific network features. In particular, the formation of hubs and modules requires an anti-homeostatic mechanism, able to associate different specific inputs between them.

To dynamically keep neuronal activity levels in physiological ranges, different forms of synaptic plasticity are also required. In particular, homeostatic mechanisms (i.e., synaptic scaling) should intervene to prevent instability induced by Hebbian plasticity [32,33]. Synaptic scaling is characterized by an increase (upscaling) or decrease (downscaling) of synaptic excitability that is mediated by change in the expression of α-amino-3-hydroxy-5-methyl-4-isoxazolepropionic acid receptors (AMPARs). Unlike LTP, synaptic scaling lacks input-specificity and is a hetero-synaptic form of plasticity. Scaling has a negative feedback action and represents a compensatory mechanism devoted to stabilizing synaptic excitability in response to persistent increase or decrease of excitatory inputs to a neuron [32,34]. At a network level, homeostatic plasticity mechanisms could therefore contribute to stabilizing network activity, and might be particularly suitable to prevent excessive hyperconnectivity in the peripheral nodes of a network.

Anti-homeostatic and homeostatic plasticity cooperate to promote optimal brain network topology and are involved in symptom compensation and remodeling after brain damage [35,36].

## 3. Inflammation Alters Synaptic Transmission and Plasticity in MS

Several mediators released during neuroinflammation can affect synaptic functioning, influencing the disease course of MS [37]. Specific proinflammatory cytokines, including interleukin (IL)-1β and tumor necrosis factor (TNF), promote excitotoxic neuronal damage in EAE, exacerbating glutamatergic transmission and reducing GABAergic signaling [6,38]. A similar set of proinflammatory cytokines could also alter synaptic functioning in human MS, as CSF collected from MS patients in the acute phase of disease reproduces the neurophysiological alterations observed in EAE when incubated in vitro on control mouse slices [39].

### 3.1. Inflammation and LTP

Studies in EAE and in patients with MS pointed out that LTP and LTD expression is affected by inflammation [40,41,42,43]. In relapsing MS patients, reduced LTP-like plasticity in response to the intermittent theta burst stimulation protocol has been reported [40]. Moreover, a synaptic plasticity deficit was normalized after three-month treatment with interferon beta 1a, suggesting a direct role of acute inflammation [40]. Specific proinflammatory molecules, including IL-1β and IL-6, influence LTP induction in vitro both in physiological and pathological conditions [44,45,46,47,48,49]. IL-1β has been consistently involved in the pathogenesis of synaptic plasticity alterations in EAE [39,41,42,43]; however, contrasting data exist on how this cytokine affects LTP and LTD expression. While it has been reported that IL-1β impairs LTP induction in mice hippocampal slices [42], enhanced LTP expression has also been observed in response to IL-1β [43]. Accordingly, in RR-MS patients the CSF levels of IL-1β have been correlated with a paradoxical LTP-like effect in response to an LTD-inducing TMS protocol [41], confirming that this cytokine also produces a profound subversion of synaptic plasticity in human MS. Similarly, IL-6 has been associated to impaired LTP induction in vitro and in MS [49]. In RR-MS patients, the CSF levels of this cytokine were correlated to impaired LTP-like plasticity explored with TMS [49]. Notably, in the same study, higher CSF levels of IL-6 were also associated with enhanced clinical expression of new brain lesions, suggesting that CSF inflammation impairs resilience to damage.

It has been shown that inflammation in MS is associated with altered Amyloid-β (Aβ) homeostasis. One study evidenced in a group of RR-MS patients a negative correlation between the number of Gd+ lesions at MRI and Aβ1-42 CSF levels [50]. Furthermore, a negative correlation has been found between the CSF concentrations of Aβ1-42 and LTP-like responses to TMS [50]. In addition, it has been reported that the CSF levels of different proinflammatory molecules negatively correlate with Aβ1-42 concentrations [49,51]. Overall, these data suggest that in MS Aβ homeostasis is as a key factor bridging inflammation, plasticity and neurodegeneration.

### 3.2. Inflammation and Upscaling

Experimental studies have shown that TNF is involved in inducing and maintaining synaptic upscaling in different brain areas [52,53,54,55]. The specific role of TNF in promoting pathologically enhanced synaptic upscaling has been elucidated in EAE mice. In particular, TNF has been associated with increased expression of AMPARs and exacerbated glutamatergic excitatory post-synaptic currents [5]. These alterations preceded the onset of clinical deficits [5] and were prevented by pre-incubation with TNF inhibitors [56]. The pathogenic role of TNF has been associated particularly with progressive MS [57,58]. It has been shown that the CSF collected from patients with progressive disease course is able to induce TNF-mediated upscaling and excitotoxic neuronal damage in vitro, thus suggesting a link between CNS inflammation and neurodegeneration [57].

These data show that inflammation in MS is associated with a profound alteration of synaptic plasticity mainly characterized by impaired LTP and overexpressed synaptic upscaling. This evidence could be crucial for shedding light on the connectivity alterations and brain network reorganization found in MS patients.

## 4. Inflammation and Brain Network Organization in MS

### 4.1. Inflammation Alters Brain Connectivity in MS

A number of MRI studies have described alterations of structural and functional connectivity (SC and FC) in MS patients [15,59,60,61,62,63,64,65,66]. Altered connectivity has already been observed in the early phases of MS [65,67,68,69], and has been correlated with clinical and cognitive deficits [15,65,70], suggesting that this approach could be more sensitive than conventional MRI in investigating early alterations [71].

The nature of connectivity changes in MS is still debated [72]. In MS patients, reduced SC and FC in different networks has been related to both white matter lesion load and cortical atrophy [73,74,75,76], though connectivity alterations have also been reported independently of structural brain damage [64,68,69]. In patients with early RR-MS, Faivre and colleagues [68] have found increased FC in different resting state networks not correlated with lesion load. Furthermore, one study exploring FC in RR-MS and CIS patients has shown reduced efficiency in the left Rolandic operculum, insula and superior temporal gyrus bilaterally, not correlated to structural damage [69]. The role of other causes of disconnection should also be considered, including the presence of diffuse microstructural damage occurring in normal appearing white matter [77,78]. Rs-fMRI studies in patients with clinically isolated syndrome (CIS) have consistently shown that altered connectivity can be found independently of demyelinating lesions or neuronal atrophy, in fact widespread functional connectivity changes have been reported in both isolated optic neuritis and CIS without brain lesions [64,79]. In particular, acute optic neuritis has been associated with reduced FC in the visual system and altered connectivity between visual and non-visual networks, indicating rapid connectivity changes in response to focal inflammation [79]; in addition, both reduced and increased FC has been reported in CIS patients without brain lesions [64], suggesting that CNS inflammation may represent a prominent cause of connectivity dysfunction in MS [7].

In line with these findings, it has been reported that not only CNS-confined inflammation, but also systemic inflammation may alter brain activity and network reorganization [80,81]. A study using positron-emission tomography has revealed that administration of endotoxin in healthy subjects was associated with increased serum levels of inflammatory molecules and enhanced fatigue and depression [80]. These findings were associated with increased and reduced glucose metabolism in the insula and anterior cingulate cortex, respectively [80]. Similarly, it has been reported that interferon-alpha administration produced mood alterations associated with a rapid decrease in overall brain network efficiency and reduced connectivity of the nucleus accumbens, thalamus and inferior temporal cortex [81]. Moreover, experimental inflammation induced by lipopolysaccharide injection has been associated with connectivity alterations in healthy subjects [82,83] and the specific role of peripheral inflammatory cytokines has been evidenced in task-based and rs-fMRI studies [80,84,85,86]. Accordingly, it has been reported that serum concentrations of IL-6, a marker of peripheral inflammation, covaried with FC in the default mode network and with connectivity in the anterior cingulate cortex and in the medial prefrontal cortex [86].

These findings suggest that central inflammation, in addition to demyelination and atrophy, could alter brain connectivity in MS.

### 4.2. Brain Network Reorganization in MS

The physiological meaning of changes in brain FC and SC during the course of MS is still debated, as both decreased and increased connectivity in different brain networks have been described [66,68,74,87]. Furthermore, both positive and negative correlations have been reported between connectivity changes and clinical measures [67,68]. Differences between studies, concerning MS phenotype, degree of disability, presence of clinical activity, and MRI measures, could be partly responsible for these apparently contrasting findings. In addition, the presence of brain network reorganization, either compensatory or maladaptive, represents an additional source of variability between data. As optimal brain network reorganization requires efficient plasticity, synaptic plasticity dysfunction observed in MS, in particular reduced LTP expression and pathologically increased synaptic upscaling, could specifically contribute to altering brain network architecture and impairing reorganization after damage.

Increased synchronization in different resting state networks has been described in CIS patients compared with both RR-MS patients and controls, suggesting the existence of early brain reorganization to damage, which is lost with disease progression [67]. However, as increased connectivity in early MS patients has been associated with higher disability, the implications of FC changes should be carefully considered [68]. Studies exploring SC in CIS and MS have been shown to strengthen local connectivity from the early phases of the disease [66,88]. In particular, increased clustering coefficient and modularity have been described in MS patients during the first year of disease, largely independent of clinical activity [66,89]. It has been proposed that such reorganization cannot be simply considered a result of structural damage, but rather represents an adaptive phenomenon [66]. Accordingly, modularization and increased local processing improve the network’s ability to react to attacks [90,91].

An extreme redistribution of both functional and structural brain connectivity, with hub loss and formation of new hubs has been reported in the early phases of MS [63,92]. A study using rs-fMRI evidenced connectivity loss in hub regions including the precuneus, cingulate cortex and frontal areas, and newly formed hubs in the left temporal pole and cerebellum [63]. Using DTI, reduced connection strength in the rich club has been demonstrated in RR-MS patients and has been correlated with cognitive impairment [92]. In particular, altered rich club and local connectivity were not found to be related to white matter damage [92]. Notably, in CIS patients rich club connectivity was preserved and alterations mostly involved feeders connecting rich club to non-rich club nodes and local connections [92]. One rs-fMRI study explored brain network reorganization in a group of CIS patients at baseline and after a one year follow-up [93] by calculating the hub disruption index, a useful and reliable measure of large-scale networks reorganization [94,95]. With such an approach, a significant hub reorganization was detected at baseline and even more after one year, showing the occurrence of brain regions with reduced and increased connectivity at the same time [93]. At baseline, increased connectivity in the right middle temporal gyrus was observed in CIS; moreover, after a one year follow-up, both a higher degree in the hippocampi, cingulate cortex and in the left parieto-occipital sulcus, and a reduced degree in the middle occipital gyrus and left posterior segment of the lateral fissure were observed. Hub reorganization correlated with better cognitive scores; conversely, no significant correlations were reported with clinical scores and white matter lesions [93].

### 4.3. Altered Synaptic Plasticity Impairs Brain Network Remodeling in MS

Inconsistencies among studies exist, but taken together they suggest that a precise pattern of global brain network reorganization can be found in MS from the early phases, characterized by reduced hub connections and increased local connectivity. Some of these alterations may represent network reorganization mediated by synaptic plasticity mechanisms, whereas others may be consequence of increasing brain damage. Altered synaptic plasticity could represent an additional mechanism contributing to lessening brain network reorganization in MS. Some key properties of synaptic plasticity share analogies with specific characteristics of neuronal networks supporting this hypothesis. Input-specificity, activity-dependency, associativity and tendency to induce synaptic instability make LTP well-suited to generate highly connected nodes and could be involved in hub formation and reorganization. Hence, reduced LTP expression could specifically impair hub remodeling, contributing to hub disruption observed in MS. Furthermore, homeostatic synaptic plasticity allows adaptation to damage promoting local and generalized connectivity increases, which could be aimed at restoring network functionality, is also likely involved in limiting the connectivity of peripheral nodes, contributing to maintaining overall excitability and wiring cost around optimal levels. Altered homeostatic plasticity may therefore promote diffuse network hyperconnectivity.

Notably, progressive MS represents a useful model to test this hypothesis. Accordingly, in progressive MS, reduced rich club connectivity and a relative increase of local connectivity have been demonstrated [96]. Consistently, absent LTP induction and pathologically enhanced synaptic upscaling have been revealed in this MS phenotype [57,97,98].

## 5. Conclusions

**Figure 1:**
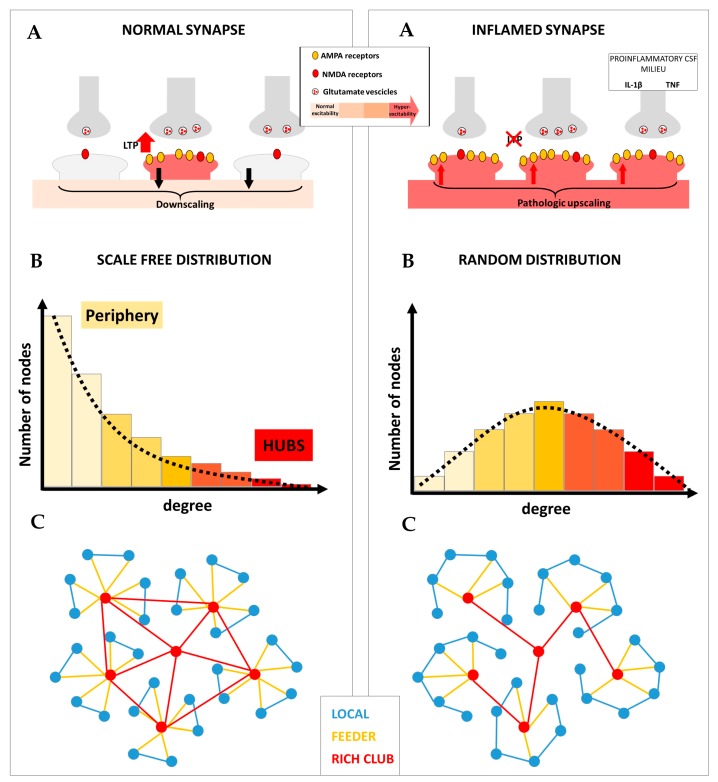
Different forms of synaptic plasticity cooperate to promote optimal brain network topology. **Left panel:** (**A**) In physiological conditions, the balance between anti-homeostatic and homeostatic plasticity allows the generation of potentiated synapses, associated with selective information processing, and prevents uncontrolled hyperexcitability. (**B**) Long-term potentiation (LTP) may be specifically involved in generating highly connected nodes (hubs); conversely, synaptic downscaling may be useful for maintaining low connectivity in the peripheral nodes of the network. The fine-tuning between these two forms of synaptic plasticity is required to form brain networks characterized by a scale-free degree distribution. (**C**) The resulting brain network architecture is characterized by elevated efficiency of information processing and resilience to random damage. **Right panel:** (**A**) In multiple sclerosis (MS), neuroinflammation is associated with impaired LTP and pathologically overexpressed synaptic upscaling, leading to uncontrolled neuronal hyperexcitability. (**B**) Disrupted LTP may selectively reduce hub connectivity, while overexpressed upscaling may contribute to increasing connectivity in the periphery. This is associated with loss of optimal brain network architecture as demonstrated by further random degree distribution. (**C**) Loss of LTP may selectively disrupt hub connectivity and rich club organization. Conversely, pathologic upscaling may promote increased local connectivity. The resulting brain network architecture dramatically reduces efficiency and impairs the ability to compensate for ongoing brain damage in MS.

The appropriate interplay between anti-homeostatic and homeostatic plasticity allows the formation of brain networks provided with peculiar characteristics associated with high resilience to damage, including scale-free degree distribution, modularity and rich club organization (Figure 1, left panel). We propose that altered synaptic plasticity in MS may promote both loss of hub connections and increased local connectivity, leading to random brain network architecture and impaired remodeling in response to damage (Figure 1, right panel). Therefore, inflammation may critically reduce brain network resilience in MS, promoting a worse disease course. This is in line with preclinical and clinical studies showing that the inflammatory CSF milieu critically influences MS disability progression and impairs clinical compensation of ongoing brain damage [49]. Conversely, it has been demonstrated that, in MS, anti-inflammatory molecules and neurotrophic factors may concur to promote a stable disease course [99]. Accordingly, the platelet-derived growth factor has been found to be absent in the CSF of patients with progressive MS, possibly contributing to explaining the impaired LTP-like plasticity expression [97]. Overall, these observations suggest that contrasting central inflammation could limit the functional disconnection and promote efficient brain network reorganization in MS.

Further studies are required to better define the relationship between CSF inflammation, synaptic plasticity and brain network structural and functional connectivity. In particular, studies combining neurophysiological investigations and fMRI measures could help to clarify the significance of brain network reorganization occurring in MS.

## Abbreviations

Aβ	amyloid-β
AMPA	α-amino-3-hydroxy-5-methyl-4-isoxazolepropionic acid
CNS	central nervous system
CSF	cerebrospinal fluid
CIS	clinically isolated syndrome
DTI	diffusion tensor imaging
EAE	experimental autoimmune encephalomyelitis
FC	functional connectivity
fMRI	functional MRI
IL	interleukin
K	node degree
LTD	long-term depression
LTP	long-term potentiation
MS	multiple sclerosis
NMDA	N-methyl-D-aspartate
PK	degree distribution
RR	relapsing-remitting
rs-fMRI	resting state-functional magnetic resonance imaging
SC	structural connectivity
TMS	transcranial magnetic stimulation
TNF	tumor necrosis factor

## References

[1] Polman C.H., Reingold S.C., Banwell B., Clanet M., Cohen J.A., Filippi M., Fujihara K., Havrdova E., Hutchinson M., Kappos L. (2015). Diagnostic criteria for multiple sclerosis: 2010 Revisions to the McDonald criteria. Ann. Neurol..

[2] Barkhof F. (2002). The clinico-radiological paradox in multiple sclerosis revisited. Curr. Opin. Neurol..

[3] Rossi S., Studer V., Motta C., Germani G., Macchiarulo G., Buttari F., Mancino R., Castelli M., De Chiara V., Weiss S. (2014). Cerebrospinal fluid detection of interleukin-1b in phase of remission predicts disease progression in multiple sclerosis. J. Neuroinflammation.

[4] Stampanoni Bassi M., Iezzi E., Landi D., Monteleone F., Gilio L., Simonelli I., Musella A., Mandolesi G., De Vito F., Furlan R. (2018). Delayed treatment of MS is associated with high CSF levels of IL-6 and IL-8 and worse future disease course. J. Neurol..

[5] Centonze D., Muzio L., Rossi S., Cavasinni F., De Chiara V., Bergami A., Musella A., D’Amelio M., Cavallucci V., Martorana A. (2009). Inflammation triggers synaptic alteration and degeneration in experimental autoimmune encephalomyelitis. J. Neurosci..

[6] Mandolesi G., Musella A., Gentile A., Grasselli G., Haji N., Sepman H., Fresegna D., Bullitta S., De Vito F., Musumeci G. (2013). Interleukin-1β alters glutamate transmission at purkinje cell synapses in a mouse model of multiple sclerosis. J. Neurosci..

[7] Stampanoni Bassi M., Gilio L., Buttari F., Maffei P., Marfia G.A., Restivo D.A., Centonze D., Iezzi E. (2017). Remodeling Functional Connectivity in Multiple Sclerosis: A Challenging Therapeutic Approach. Front. Neurosci..

[8] Achard S., Bullmore E. (2007). Efficiency and cost of economical brain functional networks. PLoS Comput. Biol..

[9] Hillary F.G., Grafman J.H. (2017). Injured Brains and Adaptive Networks: The Benefits and Costs of Hyperconnectivity. Trends. Cogn. Sci..

[10] Centonze D., Rossi S., Tortiglione A., Picconi B., Prosperetti C., De Chiara V., Bernardi G., Calabresi P. (2007). Synaptic plasticity during recovery from permanent occlusion of the middle cerebral artery. Neurobiol. Dis..

[11] Di Lazzaro V., Profice P., Pilato F., Capone F., Ranieri F., Pasqualetti P., Colosimo C., Pravatà E., Cianfoni A., Dileone M. (2010). Motor cortex plasticity predicts recovery in acute stroke. Cereb. Cortex.

[12] Watts D.J., Strogatz S.H. (1998). Collective dynamics of ‘small-world’ networks. Nature.

[13] Sporns O., Tononi G., Kötter R. (2005). The human connectome: A structural description of the human brain. PLoS Comput. Biol..

[14] Stam C.J., Reijneveld J.C. (2007). Graph theoretical analysis of complex networks in the brain. Nonlinear Biomed. Phys..

[15] Bullmore E., Sporns O. (2009). Complex brain networks: Graph theoretical analysis of structural and functional systems. Nat. Rev. Neurosci..

[16] Stam C.J. (2014). Modern network science of neurological disorders. Nat. Rev. Neurosci..

[17] Rossini P.M., Di Iorio R., Bentivoglio M., Bertini G., Ferreri F., Gerloff C., Ilmoniemi R.J., Miraglia F., Nitsche M.A., Pestilli F. (2019). Methods for analysis of brain connectivity: An IFCN-sponsored review. Clin. Neurophysiol..

[18] Biswal B.B., Yetkin F.Z., Haughton V.M., Hyde J.S. (1995). Functional connectivity in the motor cortex of resting human brain using echo-planar MRI. Magn. Reson. Med..

[19] Fox M.D., Raichle M.E. (2007). Spontaneous fluctuations in brain activity observed with functional magnetic resonance imaging. Nat. Rev. Neurosci..

[20] Sporns O., Honey C.J., Kötter R. (2007). Identification and classification of hubs in brain networks. PLoS ONE.

[21] Van den Heuvel M.P., Sporns O. (2013). An anatomical substrate for integration among functional networks in human cortex. J. Neurosci..

[22] Barabasi A.L., Albert R. (1999). Emergence of scaling in random networks. Science.

[23] Achard S., Salvador R., Whitcher B., Suckling J., Bullmore E. (2006). A resilient, low-frequency, small-world human brain functional network with highly connected association cortical hubs. J. Neurosci..

[24] Crossley N.A., Mechelli A., Scott J., Carletti F., Fox P.T., McGuire P., Bullmore E.T. (2014). The hubs of the human connectome are generally implicated in the anatomy of brain disorders. Brain.

[25] Turrigiano G.G., Nelson S.B. (2004). Homeostatic plasticity in the developing nervous system. Nat. Rev. Neurosci..

[26] Citri A., Malenka R.C. (2008). Synaptic plasticity: Multiple forms, functions, and mechanisms. Neuropsychopharmacology.

[27] Bliss T.V., Lømo T. (1973). Long-lasting potentiation of synaptic transmission in the dentate area of the anaesthetized rabbit following stimulation of the perforant path. J. Physiol..

[28] Mori F., Kusayanagi H., Nicoletti C.G., Weiss S., Marciani M.G., Centonze D. (2014). Cortical plasticity predicts recovery from relapse in multiple sclerosis. Mult. Scler..

[29] Bliss T.V., Collingridge G.L. (1993). A synaptic model of memory: Long-term potentiation in the hippocampus. Nature.

[30] Harris E.W., Ganong A.H., Cotman C.W. (1984). Long-term potentiation in the hippocampus involves activation of N-methyl-D-aspartate receptors. Brain Res..

[31] Malenka R.C., Bear M.F. (2004). LTP and LTD: An embarrassment of riches. Neuron.

[32] Turrigiano G.G., Leslie K.R., Desai N.S., Rutherford L.C., Nelson S.B. (1998). Activity-dependent scaling of quantal amplitude in neocortical neurons. Nature.

[33] Turrigiano G.G., Nelson S.B. (2000). Hebb and homeostasis in neuronal plasticity. Curr. Opin. Neurobiol..

[34] Lissin D.V., Gomperts S.N., Carroll R.C., Christine C.W., Kalman D., Kitamura M., Hardy S., Nicoll R.A., Malenka R.C., von Zastrow M. (1998). Activity differentially regulates the surface expression of synaptic AMPA and NMDA glutamate receptors. Proc. Natl. Acad. Sci. USA.

[35] Desai N.S., Cudmore R.H., Nelson S.B., Turrigiano G.G. (2002). Critical periods for experience-dependent synaptic scaling in visual cortex. Nat. Neurosci..

[36] Turrigiano G.G. (2012). Homeostatic synaptic plasticity: Local and global mechanisms for stabilizing neuronal function. Cold. Spring Harb. Perspect. Biol..

[37] Stampanoni Bassi M., Mori F., Buttari F., Marfia G.A., Sancesario A., Centonze D., Iezzi E. (2017). Neurophysiology of synaptic functioning in multiple sclerosis. Clin. Neurophysiol..

[38] Rossi S., Studer V., Motta C., De Chiara V., Barbieri F., Bernardi G., Centonze D. (2012). Inflammation inhibits GABA transmission in multiple sclerosis. Mult. Scler..

[39] Rossi S., Furlan R., De Chiara V., Motta C., Studer V., Mori F., Musella A., Bergami A., Muzio L., Bernardi G. (2012). Interleukin-1 β causes synaptic hyperexcitability in multiple sclerosis. Ann. Neurol..

[40] Mori F., Kusayanagi H., Buttari F., Centini B., Monteleone F., Nicoletti C.G., Bernardi G., Verdun Di Cantogno E., Marciani M.G., Centonze D. (2012). Early treatment with high-dose interferon beta-1a reverses cognitive and cortical plasticity deficits in multiple sclerosis. Funct. Neurol..

[41] Mori F., Nisticò R., Mandolesi G., Piccinin S., Mango D., Kusayanagi H., Berretta N., Bergami A., Gentile A., Musella A. (2014). Interleukin-1β promotes long-term potentiation in patients with multiple sclerosis. Neuromolecular. Med..

[42] Di Filippo M., Chiasserini D., Gardoni F., Viviani B., Tozzi A., Giampà C., Costa C., Tantucci M., Zianni E., Boraso M. (2013). Effects of central and peripheral inflammation on hippocampal synaptic plasticity. Neurobiol. Dis..

[43] Nisticò R., Mango D., Mandolesi G., Piccinin S., Berretta N., Pignatelli M., Feligioni M., Musella A., Gentile A., Mori F. (2013). Inflammation subverts hippocampal synaptic plasticity in experimental multiple sclerosis. PLoS ONE.

[44] Bellinger F.P., Madamba S.G., Campbell I.L., Siggins G.R. (1995). Reduced long-term potentiation in the dentate gyrus of transgenic mice with cerebral overexpression of interleukin-6. Neurosci. Lett..

[45] Li A.J., Katafuchi T., Oda S., Hori T., Oomura Y. (1997). Interleukin-6 inhibits long-term potentiation in rat hippocampal slices. Brain. Res..

[46] Schneider H., Pitossi F., Balschun D., Wagner A., del Rey A., Besedovsky H.O. (1998). A neuromodulatory role of interleukin- 1β in the hippocampus. Proc. Natl. Acad. Sci. USA.

[47] Coogan A.N., O’Neill L.A., O’Connor J.J. (1999). The P38 mitogen-activated protein kinase inhibitor SB203580 antagonizes the inhibitory effects of interleukin-1β on long-term potentiation in the rat dentate gyrus in vitro. Neuroscience.

[48] Avital A., Goshen I., Kamsler A., Segal M., Iverfeldt K., Richter-Levin G., Yirmiya R. (2003). Impaired interleukin-1 signaling is associated with deficits in hippocampal memory processes and neural plasticity. Hippocampus.

[49] Stampanoni Bassi M., Iezzi E., Mori F., Simonelli I., Gilio L., Buttari F., Sica F., De Paolis N., Mandolesi G., Musella A. (2019). Interleukin-6 Disrupts Synaptic Plasticity and Impairs Tissue Damage Compensation in Multiple Sclerosis. Neurorehab. Neural. Rep..

[50] Mori F., Rossi S., Sancesario G., Codecà C., Mataluni G., Monteleone F., Buttari F., Kusayanagi H., Castelli M., Motta C. (2011). Cognitive and cortical plasticity deficits correlate with altered amyloidβ CSF levels in multiple sclerosis. Neuropsychopharmacology.

[51] Stampanoni Bassi M., Garofalo S., Marfia G.A., Gilio L., Simonelli I., Finardi A., Furlan R., Sancesario G.M., Di Giandomenico J., Storto M. (2017). Amyloid-β Homeostasis Bridges Inflammation, Synaptic Plasticity Deficits and Cognitive Dysfunction in Multiple Sclerosis. Front. Mol. Neurosci..

[52] Stellwagen D., Malenka L.C. (2006). Synaptic scaling mediated by glial TNF-α. Nature.

[53] Becker D., Deller T., Vlachos A. (2015). Tumor necrosis factor (TNF)-receptor 1 and 2 mediate homeostatic synaptic plasticity of denervated mouse dentate granule cells. Sci. Rep..

[54] Lewitus G.M., Pribiag H., Duseja R., St-Hilaire M., Stellwagen D. (2014). An adaptive role of TNFα in the regulation of striatal synapses. J. Neurosci..

[55] Ren W., Lui Y., Zhou L., Li W., Zhong Y., Pang R., Xin W., Wei X., Wang J., Zhuet H. (2011). Peripheral nerve injury leads to working memory deficits and dysfunction of the hippocampus by upregulation of TNF-α in rodents. Neuropsychopharmacology.

[56] Haji N., Mandolesi G., Gentile A., Sacchetti L., Fresegna D., Rossi S., Musella A., Sepman H., Motta C., Studer V. (2012). TNF-α-mediated anxiety in a mouse model of multiple sclerosis. Exp. Neurol..

[57] Rossi S., Motta C., Studer V., Barbieri F., Buttari F., Bergami A., Sancesario G., Bernardini S., De Angelis G., Martino G. (2014). Tumor necrosis factor is elevated in progressive multiple sclerosis and causes excitotoxic neurodegeneration. Mult. Scler..

[58] Valentin-Torres A., Savarin C., Hinton D.R., Phares T.W., Bergmann C.C., Stohlman S.A. (2016). Sustained TNF production by central nervous system infiltrating macrophages promotes progressive autoimmune encephalomyelitis. J. Neuroinflamm..

[59] He Y., Dagher A., Chen Z., Charil A., Zijdenbos A., Worsley K., Evans A. (2009). Impaired small-world efficiency in structural cortical networks in multiple sclerosis associated with white matter lesion load. Brain.

[60] Filippi M., van den Heuvel M.P., Fornito A., He Y., Hulshoff Pol H.E., Agosta F., Comi G., Rocca M.A. (2013). Assessment of system dysfunction in the brain through MRI-based connectomics. Lancet Neurol..

[61] Schoonheim M.M., Geurts J.J., Landi D., Douw L., van der Meer M.L., Vrenken H., Polman C.H., Barkhof F., Stam C.J. (2013). Functional connectivity changes in multiple sclerosis patients: A graph analytical study of MEG resting state data. Hum. Brain Mapp..

[62] Tewarie P., Schoonheim M.M., Stam C.J., van der Meer M.L., van Dijk B.W., Barkhof F., Polman C.H., Hillebrand A. (2014). Cognitive and clinical dysfunction, altered MEG resting-state networks and thalamic atrophy in multiple sclerosis. PLoS ONE.

[63] Rocca M.A., Valsasina P., Meani A., Falini A., Comi G., Filippi M. (2016). Impaired functional integration in multiple sclerosis: A graph theory study. Brain Struct. Funct..

[64] Liu Y., Dai Z., Duan Y., Huang J., Ren Z., Liu Z., Dong H., Shu N., Vrenken H., Wattjes M.P. (2016). Whole brain functional connectivity in clinically isolated syndrome without conventional brain MRI lesions. Eur. Radiol..

[65] Shu N., Duan Y., Xia M., Schoonheim M.M., Huang J., Ren Z., Sun Z., Ye J., Dong H., Shi F. (2016). Disrupted topological organization of structural and functional brain connectomes in clinically isolated syndrome and multiple sclerosis. Sci. Rep..

[66] Fleischer V., Radetz A., Ciolac D., Muthuraman M., Gonzalez-Escamilla G., Zipp F., Groppa S. (2019). Graph Theoretical Framework of Brain Networks in Multiple Sclerosis: A Review of Concepts. Neuroscience.

[67] Roosendaal S.D., Schoonheim M.M., Hulst H.E., Sanz-Arigita E.J., Smith S.M., Geurts J.J., Barkhof G. (2010). Resting state networks change in clinically isolated syndrome. Brain.

[68] Faivre A., Rico A., Zaaraoui W., Crespy L., Reuter F., Wybrecht D., Soulier E., Malikova I., Confort-Gouny S., Cozzone P.J. (2012). Assessing brain connectivity at rest is clinically relevant in early multiple sclerosis. Mult. Scler..

[69] Liu Y., Wang H., Duan Y., Huang J., Ren Z., Ye J., Dong H., Shi F., Li K., Wang J. (2017). Functional brain network alterations in clinically isolated syndrome and multiple sclerosis: A graph-based connectome study. Radiology.

[70] Hawellek D.J., Hipp J.F., Lewis C.M., Corbetta M., Engel A.K. (2011). Increased functional connectivity indicates the severity of cognitive impairment in multiple sclerosis. Proc. Natl. Acad. Sci. USA.

[71] Liu Y., Duan Y., Dong H., Barkhof F., Li K., Shu N. (2018). Disrupted Module Efficiency of Structural and Functional Brain Connectomes in Clinically Isolated Syndrome and Multiple Sclerosis. Front. Hum. Neurosci..

[72] Ciccarelli O., Barkhof F., Bodini B., De Stefano N., Golay X., Nicolay K., Pelletier D., Pouwels P.J., Smith S.A., Wheeler-Kingshott C.A. (2014). Pathogenesis of multiple sclerosis: Insights from molecular and metabolic imaging. Lancet Neurol..

[73] Rocca M.A., Valsasina P., Martinelli V., Misci P., Falini A., Comi G., Filippi M. (2012). Large-scale neuronal network dysfunction in relapsing-remitting multiple sclerosis. Neurology.

[74] Rocca M.A., Valsasina P., Leavitt V.M., Rodegher M., Radaelli M., Riccitelli G.C., Martinelli V., Martinelli-Boneschi F., Falini A., Comi G. (2018). Functional network connectivity abnormalities in multiple sclerosis: Correlations with disability and cognitive impairment. Mult. Scler..

[75] Cruz-Gómez Á.J., Ventura-Campos N., Belenguer A., Ávila C., Forn C. (2014). The link between resting-state functional connectivity and cognition in MS patients. Mult. Scler..

[76] Zhou F., Zhuang Y., Gong H., Wang B., Wang X., Chen Q., Wu L., Wan H. (2014). Altered inter-subregion connectivity of the default mode network in relapsing remitting multiple sclerosis: A functional and structural connectivity study. PLoS ONE.

[77] Filippi M., Cercignani M., Inglese M., Horsfield M.A., Comi G. (2001). Diffusion tensor magnetic resonance imaging in multiple sclerosis. Neurology.

[78] Kocevar G., Stamile C., Hannoun S., Cotton F., Vukusic S., Durand-Dubief F., Sappey-Marinier D. (2016). Graph theory-based brain connectivity for automatic classification of multiple sclerosis clinical courses. Front. Neurosci..

[79] Wu G.F., Matthew R., Parks C.A.L., Ances B.M., Van Stavern G.P. (2015). An eye on brain integrity: Acute optic neuritis affects resting state functional connectivity impact of acute on on rs-fcMRI. Investig. Ophthalmol. Vis. Sci..

[80] Hannestad J., Gallezot J.D., Schafbauer T., Lim K., Kloczynski T., Morris E.D., Carson R.E., Ding Y., Cosgrove K.P. (2012). Cosgrove Endotoxin-induced systemic inflammation activates microglia:[^11^C]PBR28 positron emission tomography in nonhuman primates. Neuroimage.

[81] Dipasquale O., Cooper E.A., Tibble J., Voon V., Baglio F., Baselli G., Cercignani M., Harrison N.A. (2016). Interferon-α acutely impairs whole-brain functional connectivity network architecture–A preliminary study. Brain Behav. Immun..

[82] Labrenz F., Wrede K., Forsting M., Engler H., Schedlowski M., Elsenbruch S., Benson S. (2016). Alterations in functional connectivity of resting state networks during experimental endotoxemia–an exploratory study in healthy men. Brain Behav. Immun..

[83] Lekander M., Karshikoff B., Johansson E., Soop A., Fransson P., Lundström J.N., Andreasson A., Ingvar M., Petrovic P., Axelsson J. (2016). Intrinsic functional connectivity of insular cortex and symptoms of sickness during acute experimental inflammation. Brain Behav. Immun..

[84] Capuron L., Pagnoni G., Demetrashvili M., Woolwine B.J., Nemeroff C.B., Berns G.S., Miller A.H. (2005). Anterior cingulate activation and error processing during interferon-alpha treatment. Biol. Psychiatry.

[85] Harrison N.A., Brydon L., Walker C., Gray M.A., Steptoe A., Dolan R.J., Critchley H.D. (2009). Neural origins of human sickness in interoceptive responses to inflammation. Biol. Psychiatry.

[86] Marsland A.L., Kuan D.C.H., Sheu L.K., Krajina K., Kraynak T.E., Manuck S.B., Gianaros P.J. (2017). Systemic inflammation and resting state connectivity of the default mode network. Brain Behav. Immun..

[87] Tona F., Petsas N., Sbardella E., Prosperini L., Carmellini M., Pozzilli C., Pantano P. (2014). Multiple sclerosis: Altered thalamic resting-state functional connectivity and its effect on cognitive function. Radiology.

[88] Muthuraman M., Fleischer V., Kolber P., Luessi F., Zipp F., Groppa S. (2016). Structural brain network characteristics can differentiate CIS from early RRMS. Front. Neurosci..

[89] Fleischer V., Koirala N., Droby A., Gracien R.M., Deichmann R., Ziemann U., Meuth S.G., Muthuraman M., Zipp F., Groppa S. (2019). Longitudinal cortical network reorganization in early relapsing-remitting multiple sclerosis. Ther. Adv. Neurol. Disord..

[90] Latora V., Marchiori M. (2001). Efficient behavior of small-world networks. Phys. Rev. Lett..

[91] Kashtan N., Alon U. (2005). Spontaneous evolution of modularity and network motifs. Proc. Natl. Acad. Sci. USA.

[92] Shu N., Duan Y., Huang J., Ren Z., Liu Z., Dong H., Barkhof F., Li K., Liu Y. (2018). Progressive brain rich-club network disruption from clinically isolated syndrome towards multiple sclerosis. Neuroimage Clin..

[93] Koubiyr I., Deloire M., Besson P., Coupé P., Dulau C., Pelletier J., Tourdias T., Audoin B., Brochet B., Ranjeva J.P. (2018). Longitudinal study of functional brain network reorganization in clinically isolated syndrome. Mult. Scler..

[94] Termenon M., Achard S., Jaillard A., Delon-Martin C. (2016). The “Hub Disruption Index,” a Reliable Index Sensitive to the Brain Networks Reorganization. A Study of the Contralesional Hemisphere in Stroke. Front. Comput. Neurosci..

[95] Achard S., Delon-Martin C., Vértes P.E., Renard F., Schenck M., Schneider F., Heinrich C., Kremer S., Bullmore E.T. (2012). Hubs of brain functional networks are radically reorganized in comatose patients. Proc. Natl. Acad. Sci. USA.

[96] Stellmann J.P., Hodecker S., Cheng B., Wanke N., Young K.L., Hilgetag C., Gerloff C., Heesen C., Thomalla G., Siemonsen S. (2017). Reduced rich-club connectivity is related to disability in primary progressive MS. Neurol. Neuroimmunol. Neuroinflamm..

[97] Mori F., Rossi S., Piccinin S., Motta C., Mango D., Kusayanagi H., Bergami A., Studer V., Nicoletti C.G., Buttari F. (2013). Synaptic plasticity and PDGF signaling defects underlie clinical progression in multiple sclerosis. J. Neurosci..

[98] Nicoletti C.G., Monteleone F., Marfia G.A., Usiello A., Buttari F., Centonze D., Mori F. (2019). Oral D-Aspartate enhances synaptic plasticity reserve in progressive multiple sclerosis. Mult. Scler..

[99] Stampanoni Bassi M., Iezzi E., Marfia G.A., Simonelli I., Musella A., Mandolesi G., Fresegna D., Pasqualetti P., Furlan R., Finardi A. (2018). Platelet-derived growth factor predicts prolonged relapse-free period in multiple sclerosis. J. Neuroinflamm..

